# Dietary Ewe’s Yogurt Intake Selectively Modulates HDL Subfractions Without Altering LDL Particle Size in Women: A Six-Week Intervention Study

**DOI:** 10.3390/nu18121933

**Published:** 2026-06-15

**Authors:** Martina Gažarová, Petra Lenártová, Jana Kopčeková, Marta Habánová, Lucia Civáňová, Mária Kijovská, Lucia Šubová

**Affiliations:** Institute of Nutrition and Genomics, Faculty of Agrobiology and Food Resources, Slovak University of Agriculture in Nitra, Trieda Andreja Hlinku 2, 949 76 Nitra, Slovakia; martina.gazarova@uniag.sk (M.G.); jana.kopcekova@uniag.sk (J.K.); marta.habanova@uniag.sk (M.H.); lucia.civanova@uniag.sk (L.C.); xkijovska@uniag.sk (M.K.); xsuboval@uniag.sk (L.Š.)

**Keywords:** fermented milk product, ewe, health, cholesterol, lipoprotein subfractions, fats

## Abstract

**Background/Objectives:** The aim of the study was to assess the metabolic response of women to a six-week consumption of full-fat sheep milk yogurt, with a focus on cardiovascular risk markers, lipid profile, and subfractions of low-density (LDL) and high-density (HDL) lipoproteins of both atherogenic and non-atherogenic nature. **Methods:** A total of 55 women were enrolled in the nutritional intervention after the application of inclusion criteria. Blood samples were collected at baseline and after the six-week intervention period. Lipoprotein subfractions were determined in serum using the Lipoprint System LDL/HDL Subfractions Kit in combination with the Lipoprint^®^ analyzer. **Results:** In the group of women over 40 years of age with overweight or obesity, without adjustment for total cholesterol, a significant increase was observed in selected HDL subfractions (intermediate HDL-6, HDL-7 and small HDL-8, HDL-9; *p* < 0.05), while the small/large HDL ratio, LDL subfractions, and mean LDL particle size remained unchanged (*p* > 0.05). A decrease in triglycerides, LDL/HDL ratio, TG/HDL ratio, and cardiovascular risk index was recorded, alongside a slight increase in LDL-C and HDL-C levels. After adjustment for total cholesterol (T-C), no significant deterioration in the lipid profile was observed in either the group with normal or elevated T-C levels; in the group with elevated T-C, only the intermediate HDL-7 subfraction increased significantly (*p* < 0.05). Despite the presence of risk-level values in some parameters already at baseline (VLDL, IDL-A, IDL-B, small HDL), no worsening was observed. **Conclusions:** Six-week consumption of full-fat sheep milk yogurt did not lead to deterioration of the lipid profile or lipoprotein subfractions. The results suggest a neutral to mildly beneficial effect on selected cardiovascular risk markers.

## 1. Introduction

The issue of human nutrition and its impact on health remains persistently relevant. The continually deteriorating health status of the global population compels us to identify critical factors contributing to this unfavorable trend, including those related to nutrition. Unhealthy dietary habits, particularly the intake of animal fats, have long been considered significant determinants of cardiovascular risk [1]. Increased consumption of saturated fatty acids (SFA) and trans fatty acids (TFA) is associated with elevated health risk, a relationship likely mediated primarily through increased plasma concentrations of LDL cholesterol and its atherogenic effects [2]. Dairy products constitute an important component of dietary recommendations, as they provide a wide range of essential nutrients; however, they are also a source of saturated fatty acids, which account for nearly two-thirds of their fat content [3,4,5]. Current guidelines recommend limiting SFA intake to less than 10% of total energy intake, although this threshold is frequently exceeded [6,7]. Traditionally, saturated fatty acids are assumed to increase cholesterol levels and, consequently, health risk. Based on findings linking SFA intake with elevated cholesterol levels and cardiovascular disease (CVD) risk, recommendations have emphasized reducing their consumption. This has led to guidance favoring low-fat dairy products over full-fat alternatives, despite limited and inconsistent evidence in this area [8]. The relationship between saturated fat intake from dairy products and CVD risk is not consistent, suggesting that the source of fat plays a significant role in determining its metabolic effects [9]. Meta-analyses indicate either no association or even an inverse relationship between the consumption of full-fat dairy products and the risk or incidence of CVD [10,11]. Dairy products represent a heterogeneous group of foods with varying composition, nutritional properties, and processing methods, all of which ultimately influence their metabolic effects. For this reason, their relationship with CVD remains inconclusive [12,13].

Considering changes in dietary habits and the growing interest in alternative dairy products replacing cow’s milk, sheep milk products—particularly yogurt—have recently become increasingly sought after. Yogurt is a long-recognized dairy product available in various textures, fat contents, and flavors [14]. Its regular consumption has been associated with numerous health benefits, including improved lactose metabolism, antimutagenic and anticarcinogenic properties, antihypertensive effects, beneficial impacts on diarrheal diseases, stimulation of the immune system, and alleviation of symptoms of inflammatory bowel diseases [15].

Sheep milk and its derived products are characterized by a higher content of short- and medium-chain fatty acids and contain one of the highest concentrations of conjugated linoleic acid [16,17,18]. This composition predisposes sheep milk products to the prevention of cardiovascular diseases through antiatherogenic and antithrombogenic effects [19]. Studies suggest that the health risks associated with saturated fats should be assessed comprehensively, and that postprandial responses are strongly influenced by the food matrix [20,21]. Differences in physical structure, composition, and interactions among nutrients and bioactive compounds in dairy products may lead to specific effects on cardiometabolic health [21,22]. It is particularly important to evaluate yogurt as a distinct dairy food due to the unique properties of its food and fat matrices. Since dairy products differ in nutrient composition and food matrix, they may also exert different effects on the modulation of cardiovascular risk factor profiles. Studies investigating the effects of consuming various animal products have generally focused on indicators such as total cholesterol, LDL-C, or HDL-C, but not on their lipoprotein subfractions. The influence of dietary fats on the lipid profile of consumers is undeniable; however, it remains highly debated whether increases in LDL-C and HDL-C, and thus total cholesterol, are associated with increased or decreased cardiovascular risk, as both types of lipoproteins contain atherogenic and non-atherogenic subfractions that may or may not elevate cardiovascular risk [23,24]. Their beneficial or harmful effects arise from their composition at the molecular level. Dyslipidemia and an unfavorable lipid profile are characterized by elevated levels of lipids and lipoproteins, i.e., total cholesterol, low-density lipoproteins, and triglycerides, as well as reduced levels of “protective” high-density lipoproteins [25,26,27]. For decades, this condition has been considered an important negative factor in the process of atherogenesis, partly because the cholesterol content in low- and high-density lipoproteins exhibited considerable variability [28,29]. However, the discovery of lipoprotein subfractions has called into question the importance and relevance of their cholesterol content [29,30]. To date, seven LDL subfractions (large LDL1, intermediate LDL2, and small LDL3–7) and ten HDL subfractions (large HDL1–3, intermediate HDL4–7, and small HDL8–10) have been identified [31]. Emerging evidence suggests that conventionally defined lipid profiles may not differ between patients with cardiovascular diseases and healthy individuals, and that the assessment of lipoprotein subfractions is likely to be more informative than the measurement of lipid and lipoprotein concentrations alone [23,24].

The aim of this study was therefore to determine the metabolic response of female consumers to the intake of full-fat yogurt made from sheep milk over a six-week period, with respect to: (i) cardiovascular risk markers, i.e., lipid profile; (ii) changes in low-density lipoprotein subfractions of both atherogenic and non-atherogenic nature; and (iii) changes in high-density lipoprotein subfractions of both atherogenic and non-atherogenic nature. To the best of our knowledge and based on available evidence, no study has yet been conducted that comprehensively evaluates changes in both types of lipoprotein subfractions in relation to the consumption of sheep milk yogurt.

## 2. Materials and Methods

### 2.1. Participants and Study Design

This study involved a 6-week nutritional intervention using full-fat sheep’s milk yogurt. Between March and May 2024, women were recruited on a voluntary basis. We sought to limit the study population to women over the age of 40. This population undergoes important hormonal and metabolic changes associated with the menopausal transition, including alterations in lipid metabolism and increased cardiovascular risk. In addition, previous studies have demonstrated gender-related differences in lipoprotein metabolism, HDL functionality, and dietary responses. By limiting the study population to women, we sought to reduce biological variability and obtain a more homogeneous cohort for evaluating the metabolic effects of sheep’s milk yogurt consumption.

After applying the inclusion and exclusion criteria, 55 female participants were selected from the initial group of 63 individuals who had expressed interest in consuming sheep’s yogurt. The following inclusion criteria were applied: acceptance of all conditions and requirements of the clinical study confirmed by written consent; voluntariness of participation; staying in the study for the entire duration of the intervention (with the exception of serious health or personal reasons that would arise during the course of the study); age over 40 years; female sex; absence of pharmacological treatment that could substantially affect lipid metabolism.

The exclusion criteria included: pregnancy or suspected pregnancy, BMI ≥ 50 kg/m^2^, acute coronary syndrome, heart failure, thyroid dysfunction, liver disease, renal insufficiency, food allergy or intolerance, contraindications to bioimpedance measurements, adherence to restrictive dietary patterns (e.g., vegetarian or vegan diets), planned weight-loss interventions, and any diagnosed severe chronic disease that could interfere with study outcomes or participant safety.

The study was conducted according to the guidelines of the Declaration of Helsinki and approved by the Slovak University of Agriculture (SUA) in Nitra, Institute of Animal Husbandry and Institute of Nutrition and Genomics, Slovak Republic, and by the ethical review board—Ethical Committee of the Specialized Hospital of St. Zoerardus Zobor in Nitra, Kláštorská 131, 94901 Nitra, Slovak Republic (study protocol No. 20242404/1). Written informed consent was obtained from all the participants prior to their involvement in the study.

### 2.2. Dietary Intervention

The dietary intervention involved the daily consumption of full-fat sheep’s milk yogurt over a six-week period during May and June 2024. The sheep were fed a standard feed mixture supplemented with a mineral lick used to enrich their diet. The yogurt was produced from sheep’s milk that had undergone pasteurization. After cooling to 42–44 °C, the milk was inoculated with a commercial yogurt starter culture containing *Lactobacillus delbrueckii* subsp. *bulgaricus* and *Streptococcus thermophilus*, as declared by the manufacturer. Fermentation proceeded at 42–44 °C for 4–6 h, after which the yogurt was stored under refrigeration at 6 ± 1 °C.

The daily intake consisted of 150 g of plain, full-fat yogurt without added flavorings. Participants consumed the yogurt on its own, without any additives or combination with other foods. It was recommended to consume it preferably as a mid-morning or afternoon snack. The nutritional composition of yogurt is presented in Table 1.

### 2.3. Anthropometric and Somatic Measurements

Body composition was assessed using multi-frequency bioelectrical impedance analysis (MF-BIA) with the InBody 970 device (Biospace Co., Ltd., Seoul, Republic of Korea). All measurements were conducted under strictly standardized conditions and performed by a single trained examiner to minimize inter-observer variability. Participants were informed about the measurement procedure in advance.

Prior to testing, participants were instructed to avoid excessive fluid intake, abstain from alcohol for 24 h, refrain from consuming foods high in sugar, salt, or fat for at least 12 h, and avoid intense physical activity for a minimum of 12 h before measurement. All participants provided written informed consent and agreed to the processing of personal data using the Lookin’Body 3.0 software (InBody Co., Ltd., Seoul, Republic of Korea).

The following parameters were directly measured using bioimpedance analysis: basal metabolic rate (BMR, kcal), height (m), body weight (kg), waist circumference (WC, cm), hip circumference (HC, cm), body fat mass (BFM, %), and visceral fat area (VFA, cm^2^). Body mass index (BMI) was calculated as weight (kg) divided by height squared (m^2^). The waist-to-hip ratio (WHR) and waist-to-height ratio (WHtR) were calculated as waist circumference (cm) divided by hip circumference (cm) and height (m), respectively [32,33,34,35].

### 2.4. Blood Sampling and Analysis of Biochemical Parameters

Blood samples were collected by trained personnel at baseline (prior to the intervention) and immediately after the six-week intervention period. All collections were carried out in the morning following an overnight fast of at least 8 h. Whole blood samples were processed by centrifugation: EDTA tubes at 1800 rpm for 15 min and serum gel tubes at 3000 rpm for 10 min at 4 °C using the Hettich MIKRO 220R (Andreas Hettich GmbH & Co., Tuttlingen, Germany). Separated serum and plasma samples were subsequently stored at −80 °C until further analysis.

To evaluate metabolic responses, the following parameters were analyzed: lipid profile (total cholesterol, T-C; low-density lipoprotein cholesterol, LDL-C; high-density lipoprotein cholesterol, HDL-C; triglycerides, TG); glucose (GLU); high-sensitivity C-reactive protein (hs-CRP); uric acid (UA); and cardiovascular disease risk (T-C/HDL-C; LDL/HDL; TG/HDL). Analyses were performed using the Biolis 24i Premium (Tokyo Boeki Machinery, Tokyo, Japan) with commercial reagents supplied by DiaSys Diagnostic Systems GmbH (Holzheim, Germany) and Randox Laboratories Ltd. (Crumlin, UK).

Reference ranges for the monitored parameters were as follows: T-C 3.0–5.0 mmol/L; LDL-C 0–3.9 mmol/L; HDL-C 1.2–2.7 mmol/L; TG 0.2–1.92 mmol/L; LDL/HDL < 3; T-C/HDL < 4 (according to NCEP ATP III) [36,37]; GLU 3.9–5.6 mmol/L; hs-CRP 0–6 mg/L; UA 154–357 µmol/L [38].

Lipoprotein subfractions, including VLDL, IDL (A, B, C), and LDL (LDL1–7), were determined in serum using the Lipoprint System LDL Subfractions Kit (Quantimetrix Corporation, Redondo Beach, CA, USA) in combination with the Lipoprint^®^ analyzer (Quantimetrix Corporation, Redondo Beach, CA, USA), following the manufacturer’s protocol. This method is based on linear electrophoresis in non-denaturing polyacrylamide gel, enabling separation and quantification of lipoprotein subfractions. Based on LDL particle size, phenotypes were classified as non-atherogenic phenotype A (>26.8 nm), intermediate phenotype AB (26.53–26.79 nm), and atherogenic phenotype B (<26.5 nm) [39].

HDL subfractions were analyzed using the Lipoprint System HDL Subfractions Kit (Quantimetrix Corp., Redondo Beach, CA, USA), also based on linear polyacrylamide gel electrophoresis. This method allows separation and quantification of up to ten HDL subfractions, which were grouped into three main categories: HDL-Large (HDL 1–3), HDL-Intermediate (HDL 4–7), and HDL-Small (HDL 8–10). Cholesterol concentrations within HDL subfractions were calculated using the Lipoware software (version M13.1) by multiplying total cholesterol concentration by the relative area under the curve of individual subfraction bands [39].

### 2.5. Statistical Analysis

Microsoft Office Excel 2016 (Los Angeles, CA, USA) in combination with XLSTAT (Version 2019) was used to process data. Statistical analysis was carried out using the STATISTICA 13 computer software (TIBCO Software, Inc., Palo Alto, CA, USA) and MedCalc ver.23.4.8 software. The normality of variable distribution was checked with the Shapiro–Wilk test. Normally distributed variables are presented as mean (standard deviation, SD), whereas non-normally distributed variables are presented as median (interquartile range, IQR). Levels of statistical significance were determined at *p* < 0.05. With a multi-factorial variance analysis (ANOVA), we tested the differences between variables and compared using Fisher’s Post Hoc Test; non-normal data we tested with the non-parametric Mann–Whitney U test. To evaluate the relationship between variables, we used Spearman’s correlation analysis.

## 3. Results

The research intervention group consisted of fifty-five women with age of 49 years (range between 40 and 67 years). According to waist circumference (88.50 cm; IQR 80.80–105.48 cm), body fat mass (33.70%; IQR 26.58–41.56%), visceral fat area (100.57 cm^2^; IQR 72.34–146.02 cm^2^), body mass index (25.00 kg/m^2^; IQR 21.85–29.60 kg/m^2^), and waist-to-hip ratio (0.91; IQR 0.88–0.99), women were mostly overweight or obese. Baseline characteristics of the participants are summarized in Table 2.

### 3.1. Post-Intervention Changes in Biochemical Parameters and LDL/HDL Subfractions

Table 3 presents the changes in biochemical parameters following six weeks of consumption of yogurt made from sheep milk. Significant changes were observed only for CRP (*p* < 0.001), calcium (*p* < 0.01), magnesium (*p* < 0.01), and sodium (*p* < 0.001). However, the values observed remained within the reference ranges and cannot be interpreted as a negative effect.

Table 4 summarizes post-intervention changes in the lipid profile in terms of LDL and HDL subfractions, as well as cardiovascular risk. Significant changes were observed only in intermediate HDL-6 (*p* < 0.05) and HDL-7 (*p* < 0.001), as well as in small HDL-8 (*p* < 0.01) and HDL-9 (*p* < 0.01), with increases in values recorded in all cases. However, the small-to-large HDL particle ratio did not change significantly (*p* > 0.05). Likewise, LDL subfractions, mean LDL particle size, and cardiovascular risk indices did not change significantly following the intervention (*p* > 0.05). Risk-related pre- and post-intervention values were observed for VLDL, IDL-A, small HDL, and the small-to-large HDL subfraction ratio; however, these parameters were not significantly affected by the intervention (*p* > 0.05).

### 3.2. Post-Intervention Changes in Biochemical Parameters and LDL/HDL Subfractions in Relation to Total Cholesterol

Table 5 summarizes post-intervention changes in biochemical parameters adjusted for total cholesterol. The cohort of women was stratified into two groups according to whether total cholesterol (T-C) levels were within the normal range (*n* = 20) or elevated (*n* = 35). In the group of women with normal T-C values, significant changes were observed only for magnesium (*p* < 0.05) and sodium (*p* < 0.05). In the group with elevated T-C, significant changes were found for CRP (*p* < 0.05) and calcium (*p* < 0.05). Between-group differences were identified for T-C (*p* < 0.001), TG (*p* < 0.01), and calcium levels (*p* < 0.01), with higher values observed in the group with elevated T-C.

Table 6 summarizes changes in LDL and HDL subfractions and cardiovascular risk following the intervention, adjusted for total cholesterol (T-C). In the group with normal T-C levels, no significant post-intervention changes were observed after six weeks of sheep milk yogurt consumption (*p* > 0.05). Risk-related pre- and post-intervention values were identified as VLDL and small HDL, although without statistical significance. In the group with elevated T-C, a significant change was observed only in the intermediate HDL-7 subfraction (*p* < 0.05). In this group, risk-related pre- and post-intervention values were also observed not only for VLDL and small HDL, but additionally for IDL-A and IDL-B. Considering between-group differences, higher values were observed in the elevated T-C group for most parameters. Post-intervention significant differences between groups were identified for LDL (*p* < 0.001), IDL-A (*p* < 0.01), IDL-B (*p* < 0.01), IDL-C (*p* < 0.001), LDL-1 (*p* < 0.01), LDL-2 (*p* < 0.01), HDL (*p* < 0.05), HDL-10 (*p* < 0.05), small HDL (*p* < 0.05), LDL/HDL (*p* < 0.01), TG/HDL (*p* < 0.05), and T-C/HDL (*p* < 0.01).

**Figure 1:**
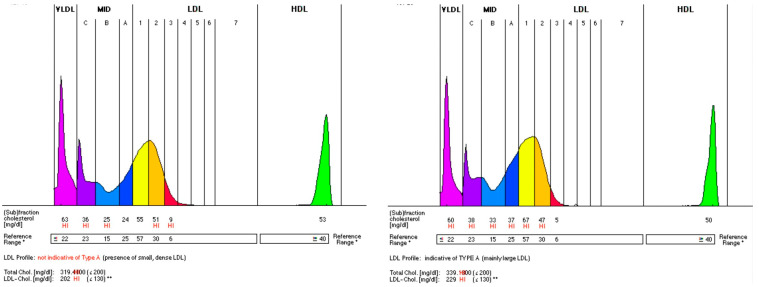
Changes in LDL subfractions in one of the participants due to a six-week intervention. * References ranges derived from 125 serum samples that met the NCEP ATP III guidelines for desirable lipid status. ** LDL-C is comprised of the sum of cholesterol in Mid bands C through A as well as all the subfractions.

**Figure 2:**
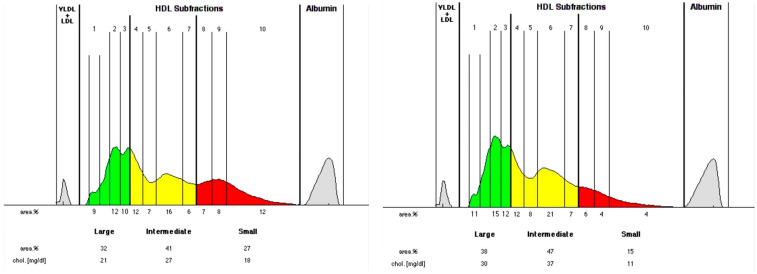
Changes in HDL subfractions in one of the participants due to a six-week intervention.

Figure 1 and Figure 2 illustrate changes in LDL and HDL subfractions in one of the participants before and after the intervention, in whom a significant improvement in the lipid profile and the distribution of lipoprotein subfractions was observed.

Table 7 presents the correlation relationships between LDL and HDL subfractions and total cholesterol, triglycerides, and cardiovascular risk. A strong correlation was confirmed between T-C and VLDL, IDL-A, IDL-B, IDL-C, LDL-C, LDL-1, LDL-2, and HDL-10. In the case of triglycerides, a strong correlation was observed with VLDL, IDL-C, LDL-C, and LDL-2, alongside a strong inverse association with large HDL and HDL-2 to HDL-5 subfractions. Cardiovascular risk indices showed strong associations with VLDL, IDL-C, and LDL-C, and inverse associations with HDL-C, intermediate HDL, large HDL, and HDL-2 to HDL-5 subfractions.

## 4. Discussion

The aim of this study was to determine how six weeks of consumption of full-fat yogurt made from sheep milk affect selected cardiovascular risk markers (lipid profile), and low- and high-density lipoprotein subfractions of both atherogenic and non-atherogenic nature. The results of the study indicate a potentially neutral (non-harmful) effect of full-fat sheep milk yogurt consumed during the six-week intervention period. Based on the findings, we focus primarily on changes in lipoprotein subfractions.

When considering the group of women over 40 years of age with overweight or obesity, without adjustment for total cholesterol, significant changes were observed only in intermediate HDL-6 and HDL-7, as well as in small HDL-8 and HDL-9, in all cases reflecting an increase in values. Importantly, the small-to-large HDL particle ratio did not change significantly, nor did LDL subfractions, mean LDL particle size, or overall cardiovascular risk. Although risk-related values were already present at baseline for VLDL, IDL-A, small HDL, and the small-to-large HDL subfraction ratio, these parameters were not significantly affected by the intervention (*p* > 0.05). Following the intervention, a 4% decrease in triglycerides (TG) was observed, alongside a 4.8% increase in LDL-C and a 4.9% increase in HDL-C. The LDL/HDL ratio decreased by 5.8%, T-C/HDL decreased by 8.6%, and the TG/HDL ratio decreased by 7%.

When the issue is viewed in the context of intervention-induced changes adjusted for T-C, no lipid parameter significantly worsened in the group of women with normal T-C levels because of the intervention. In the group with elevated T-C, only the intermediate HDL-7 subfraction showed a significant change, with an observed increase. Considering that risk-related values were already present at baseline in this group for VLDL, small HDL, IDL-A, and IDL-B, these parameters did not show any significant deterioration after the intervention. Between-group differences were more pronounced, which was expected given the baseline lipid profile values and natural differences associated with elevated T-C. This concerns LDL, IDL-A, IDL-B, IDL-C, LDL-1, LDL-2, HDL, HDL-10, and small HDL, as well as the LDL/HDL ratio, TG/HDL ratio, and T-C/HDL ratio. Despite the presence of elevated and risk-related values in some parameters, the overall findings can be interpreted positively, as regardless of baseline T-C levels, no significant deterioration in cardiovascular markers was observed either in the full cohort or in the subgroups stratified by adjusted T-C.

Numerous clinical studies indicate that the initiation and progression of atherosclerosis are determined by the number, size, and modification of LDL particles. Sniderman emphasized that it is not the total LDL-C concentration, but rather the number of LDL particles that represents a key determinant in the pathogenesis of cardiovascular diseases [40,41,42]. According to Allaire et al. [43], LDL particle size constitutes a non-causal risk factor for ischemic heart disease. Small LDL particles are more atherogenic than medium or large ones, primarily due to their prolonged plasma half-life and increased likelihood of penetrating the subendothelial space [44,45]. They exhibit lower affinity for LDL receptors and greater permeability into the arterial wall, where they bind more effectively to glycosaminoglycans [46] and are taken up by macrophages, contributing to atherosclerotic plaque formation [47]. Importantly, small dense LDL particles are more susceptible to oxidation, and their elevated levels are associated with reduced HDL concentrations and increased triglyceride levels [48].

Cardiovascular disease risk factors are generally also considered to include low levels of high-density lipoproteins. However, since HDL does not represent a homogeneous lipid fraction, an increase in HDL-C should not automatically be interpreted as a potentially protective effect [49,50,51]. The functionality and particle size of HDL, similarly to LDL, have therefore gained increasing attention [52,53,54]. Under certain conditions (oxidative stress, systemic inflammation, obesity), so-called dysfunctional HDL is formed, which may exert adverse effects on the organism [55]. According to current knowledge, large HDL particles containing apolipoproteins are considered atheroprotective [48] and are associated with lower cardiovascular risk [56]. Ezhov et al. [57] found that the concentration of intermediate HDL subfractions was inversely associated with the prevalence of coronary artery disease in middle-aged men. Similar results were reported by Muchová et al. [58] in patients with hypercholesterolemia and by Femlak et al. [59] in patients with advanced type 2 diabetes mellitus. Li et al. [60] demonstrated that high HDL levels are associated with a lower incidence of future cardiovascular events. Consequently, a simultaneous decrease in large HDL and an increase in small HDL are associated with increased cardiovascular risk. In relation to HDL-C, a novel metabolic marker has recently been proposed, based on the presence of high or low triglyceride (TG) concentrations combined with low HDL-C levels. In our study, we observed a positive trend toward a reduction in these parameters. Similar findings were reported by Taormina et al. [61] following a diet containing full-fat yogurt. Our results also support the findings of Yuan et al. [62], who demonstrated that a lower TG/HDL-C ratio was associated with a higher intake of saturated fatty acids (SFA) from dairy products.

Very low-density lipoproteins (VLDL) also act as carriers of triglycerides. Chylomicrons and larger VLDL particles are the lipoproteins with the highest triglyceride content, and an increase in these particles is associated with elevated plasma TG concentrations [63]. Through the action of lipoprotein and hepatic lipases, VLDL particles are transformed into intermediate-density lipoprotein (IDL) particles and subsequently into LDL [50]. According to Berneis and Krauss [28], when hepatic triglyceride availability is low, the liver secretes VLDL1 particles (TG-rich), which are converted into LDL-3 and IDL2 (TG-poor), and subsequently into LDL1. When TG availability is high, the liver secretes larger VLDL1 particles (TG-rich), which are converted into LDL-4, while VLDL2 particles (TG-poor) are converted into LDL2 [28]. Individuals with hypertriglyceridemia are expected to have an increased number of small LDL particles, which are relatively cholesterol-poor and triglyceride-rich compared with individuals with normal triglyceride concentrations. Accumulating evidence suggests that components of milk fat may reduce blood TG concentrations primarily through suppression of hepatic TG production. At the same time, it is hypothesized that their combination within the milk fat matrix may exert synergistic effects in reducing TG levels, which supports the need for further mechanistic research focused on this food matrix [64,65].

The fatty acid composition of sheep milk yogurt may partly explain the neutral to mildly beneficial metabolic effects observed in the present study. Although sheep milk yogurt contains a relatively high proportion of saturated fatty acids (SFAs), increasing evidence indicates that individual SFAs differ substantially in their biological effects and should not be considered a homogeneous nutrient group. In the yogurt used in our intervention, palmitic acid, myristic acid, and stearic acid represented the major saturated fatty acids. While palmitic and myristic acids have traditionally been associated with increases in circulating cholesterol concentrations, stearic acid appears to exert a largely neutral effect on plasma lipids and cardiovascular risk markers. Furthermore, sheep milk is characterized by a relatively high content of short- and medium-chain fatty acids, which are rapidly absorbed and preferentially oxidized, resulting in metabolic effects distinct from those of longer-chain fatty acids [66]. Importantly, contemporary research suggests that the cardiometabolic effects of dairy foods cannot be explained solely by their fatty acid composition. Rather, the overall dairy matrix, including interactions among fatty acids, milk proteins, minerals, phospholipids, and the milk fat globule membrane, appears to play a critical role in determining metabolic responses [67,68]. This concept is particularly relevant for fermented dairy products such as yogurt, where fermentation-derived metabolites and structural characteristics may further modify nutrient digestion, absorption, and lipid metabolism. Consequently, the health effects of fermented dairy products often differ from those predicted based solely on their saturated fat content [67]. Recent evidence from systematic reviews indicates that fermented dairy products generally exert neutral or modestly beneficial effects on blood lipids and cardiovascular health. For example, a recent systematic review by Yilmaz et al. [69] reported that yogurt consumption was associated with either neutral effects or modest improvements in lipid-related cardiometabolic markers. Similarly, observational and intervention studies have consistently shown that consumption of full-fat fermented dairy products is not associated with increased cardiovascular disease risk despite their relatively high SFA content [68,70]. These findings support the hypothesis that the absence of adverse changes in LDL particle size and LDL subfractions observed in the present study may be attributable not only to the fatty acid profile of sheep milk yogurt but also to the complex interactions within the fermented dairy matrix. Nevertheless, since the present study was not designed to investigate the specific effects of individual fatty acids, future studies incorporating detailed fatty acid profiling and lipidomic analyses are warranted to better elucidate the mechanisms underlying these observations.

In addition to its fatty acid composition, sheep milk yogurt contains several other bioactive components that may contribute to cardiovascular health. Sheep milk proteins and fermentation-derived bioactive peptides have been reported to exert antihypertensive, anti-inflammatory, and endothelial-protective effects, partly through modulation of angiotensin-converting enzyme activity and vascular function [71,72]. Furthermore, sheep milk is naturally rich in calcium, phosphorus, and magnesium, minerals that may influence lipid metabolism and cardiovascular regulation. Calcium has been suggested to reduce intestinal fat absorption and promote fecal excretion of fatty acids, thereby potentially improving blood lipid profiles [67,73]. Another potentially relevant component is the milk fat globule membrane (MFGM), a complex structure rich in phospholipids, sphingolipids, and membrane-associated proteins. Growing evidence suggests that MFGM may attenuate inflammation, improve lipid metabolism, and partly counteract the cholesterol-raising effects traditionally attributed to saturated fatty acids [68,74]. Therefore, the cardiometabolic effects of sheep milk yogurt are likely determined by the combined action of fatty acids, proteins, minerals, fermentation-derived compounds, and MFGM components rather than by saturated fatty acid content alone. This concept may partly explain why no adverse changes in LDL particle size, LDL subfractions, or cardiovascular risk indices were observed in the present study despite the consumption of a full-fat dairy product.

Despite their relatively high content of saturated fatty acids, dairy products also contain several bioactive compounds with potentially beneficial effects (e.g., medium-chain fatty acids, naturally occurring trans-fatty acids, vitamin K, and calcium) [75]. Fermented dairy products and probiotics may influence gut microbiota, thereby affecting cardiometabolic risk [76]. Probiotic-enriched dairy products exhibit a hypocholesterolemic effect through modulation of the intestinal microbiota and the promotion of fiber fermentation, leading to the production of short-chain fatty acids (SCFAs). SCFAs subsequently inhibit hepatic cholesterol synthesis and enhance its uptake. Meta-analyses consistently demonstrate that the consumption of yogurt and other probiotic-enriched dairy products leads to a reduction in total cholesterol [77,78], with observed increases in HDL-C and simultaneous decreases in LDL-C [77].

Research findings report inconsistent effects of the consumption of full-fat dairy products on consumers’ lipid profiles; however, they predominantly indicate a beneficial or neutral effect of full-fat cheese or yogurt intake on T-C, LDL-C, HDL-C, and TG concentrations [79,80]. Chen et al. [81] observed a decrease in TG concentrations following 24 weeks of full-fat yogurt consumption. Chiu et al. [82] also reported approximately 13% lower plasma TG concentrations after consumption of a diet containing full-fat dairy products (primarily milk, cheese, and yogurt) compared with an equivalent diet in which low-fat dairy products were used instead. Current recommendations for the prevention of cardiovascular diseases are largely based on the assumption that fatty acids, particularly those from dairy products, increase cholesterol levels. However, more recent evidence indicates distinct metabolic effects of individual fatty acids. Moreover, the biological effect of foods is modulated by their structure, which influences digestion and nutrient bioavailability. A modern nutritional approach therefore emphasizes the evaluation of whole foods and dietary patterns rather than isolated nutrients as the primary determinants of health [76,83,84].

Our study has certain limitations. The main limitation is the small number of participants, which may reduce statistical power. The applicability of our findings may be restricted to females, individuals of Caucasian ethnicity, and women over 40 years of age. It is evident that further studies should be conducted with larger sample sizes, in diverse population groups in terms of age and sex, as well as the presence of different cardiovascular risk factors, and with a longer intervention period. To the best of our knowledge, this is the first study among Slovak women over 40 years of age to evaluate the effect of short-term consumption of full-fat sheep milk yogurt on the lipid profile, focusing not only on LDL subfractions but also on HDL subfractions. A key strength of the study is the detailed analysis of the lipid profile in women in terms of LDL subfractions (1–7), and particularly HDL subfractions (1–10). The previously assumed negative effect of animal fats on small dense LDL particles was not confirmed in this study; however, significant changes in HDL subfractions were observed. The analysis of lipoprotein subclass concentrations and distribution is not yet a routine part of research or clinical practice; nevertheless, in our study it provided additional insights and expanded the understanding of the effects of full-fat sheep milk yogurt consumption on the lipid profile of consumers. Further studies are, of course, warranted. The beneficial effects of fermented dairy products on the lipid profile are also attributed to the beneficial bacteria present; however, in our product, the bacterial strains were identified solely according to the manufacturer’s declaration and neither the viability of the bacteria nor the functional properties were independently assessed; therefore we cannot directly attribute the observed effects to the starter culture. An additional limitation of the present study is that smoking status and alcohol consumption were not included among the analyzed covariates. Although participants were instructed to maintain their habitual lifestyle throughout the intervention period, and no substantial lifestyle changes were reported, the potential influence of these factors on lipid metabolism and cardiovascular risk markers cannot be completely excluded. Furthermore, dietary intake was not standardized or controlled during the intervention. Participants were instructed to maintain their usual dietary habits throughout the study period; therefore, the potential influence of individual dietary variability on lipid metabolism cannot be completely excluded. Future studies should include a more detailed assessment of smoking habits, alcohol intake, dietary habits and other lifestyle-related factors that may affect cardiometabolic outcomes.

## 5. Conclusions

Six-week consumption of full-fat sheep milk yogurt resulted in only limited changes in biochemical parameters, with the significant differences observed (CRP, Ca, Mg, Na) remaining within reference ranges and lacking clinical adverse relevance. The intervention did not affect LDL subfractions, mean LDL particle size, or overall cardiovascular risk. Changes in the lipid profile were selectively reflected in increases in certain HDL subfractions (HDL-6, HDL-7, HDL-8, HDL-9), without affecting the small-to-large HDL particle ratio. Despite the presence of risk-related values (VLDL, IDL, small HDL), no significant changes were observed in these parameters. Partial improvement was suggested by reductions in the LDL/HDL, TG/HDL, and T-C/HDL ratios. Stratification according to total cholesterol confirmed the absence of any adverse effect of the intervention, as no significant changes were observed in the group with normal T-C, while in the group with elevated T-C only the HDL-7 subfraction was affected. Between-group differences primarily reflected baseline lipid status.

Overall, the results suggest that short-term consumption of full-fat sheep milk yogurt exerts a neutral to mildly beneficial effect on the lipid profile, without worsening cardiovascular risk, with potential benefits mainly related to modulation of HDL subfractions. Our findings support epidemiological evidence that does not associate consumption of full-fat dairy products with adverse metabolic effects. This study represents one of the first to comprehensively evaluate changes in both types of lipoproteins subfractions in the context of sheep milk yogurt consumption. Further clinical research of this type is needed to strengthen the evidence base and to more precisely elucidate the role of milk fat in metabolic health.

## Figures and Tables

**Table 1:** Nutritional composition of sheep’s yogurt per 100 g of edible portion.

Parameters	Content	Parameters	Content
Dry matter [g/100 g]	18.17	Palmitic acid [g/100 g of fat]	27.82
Energy value [kJ/100 g]	436.8	Stearic acid [g/100 g of fat]	9.56
Fat [g/100 g]	8.1	Oleic acid [g/100 g of fat]	16.42
Cholesterol [mg/kg]	217.58	Linoleic acid [g/100 g of fat]	1.44
Saturated fatty acids [g/100 g]	6.33	Alpha-linolenic acid [g/100 g of fat]	1.01
Monounsaturated fatty acids [g/100 g]	1.63	Carbohydrates [g/100 g]	4.62
Polyunsaturated fatty acids [g/100 g]	0.17	Lactose [g/100 g]	2.71
Trans fatty acids [g/100 g]	0.11	Protein [g/100 g]	4.63
Omega-3 fatty acids [g/100 g]	0.09	Sodium [mg/kg]	559
Omega-6 fatty acids [g/100 g]	0.13	Potassium [mg/kg]	1271
Lauric acid [g/100 g of fat]	4.93	Magnesium [mg/kg]	137
Myristic acid [g/100 g of fat]	14.31	Calcium [mg/kg]	1617
		Total phosphorus [mg/kg]	1249

**Table 2:** Basic characteristics of the participants in terms of anthropometric indicators (*n* = 55).

Parameters	Med	IQR	Min	Max
Age [years]	49.00	44.00–53.00	40.00	67.00
BMR [kcal]	1398	1306–1463	1176	1618
Height [m]	1.67	1.63–1.69	1.57	1.91
Weight [kg]	67.40	61.15–83.45	46.80	100.00
BMI [kg/m^2^]	25.00	21.85–29.60	16.70	39.31
BFM [%]	33.70	26.58–41.56	18.50	50.48
VFA [cm^2^]	100.57	72.34–146.02	34.70	210.30
WC [cm]	88.50	80.80–105.48	71.60	123.60
HC [cm]	97.10	91.83–104.60	84.50	114.80
WHR	0.91	0.88–0.99	0.83	1.08
WHtR	0.54	0.48–0.62	0.40	0.78

Data are expressed as median (Med) and interquartile range (IQR); Min, minimum; Max, maximum; BMR, basal metabolic rate; BMI, body mass index; BFM, body fat mass; VFA, visceral fat area; WC, waist circumference; HC, hip circumference; WHR, waist-to-hip ratio; WHtR, waist-to-height ratio.

**Table 3:** Pre–post intervention changes in terms of biochemical indicators (*n* = 55).

Parameters	Pre				Post				*p*-Value
	Med	IQR	Min	Max	Med	IQR	Min	Max	
T-C [mmol/L]	5.50	4.70–6.35	3.48	8.26	5.67	4.99–6.41	3.55	8.77	>0.05
TG [mmol/L]	1.12	0.76–1.44	0.58	3.16	1.00	0.77–1.40	0.43	3.01	>0.05
GLU [mmol/L]	5.20	4.80–5.50	4.00	6.20	5.30	5.03–5.70	4.30	7.00	>0.05
UA [μmol/L]	253	218–323	146	459	258	221–299	147	438	>0.05
ALP [μkat/L]	1.16	0.99–1.55	0.62	2.10	1.14	0.93–1.38	0.62	2.33	>0.05
ALT [μkat/L]	0.26	0.21–0.33	0.14	0.56	0.23	0.17–0.33	0.06	0.68	>0.05
AST [μkat/L]	0.33	0.29–0.39	0.23	0.53	0.32	0.29–0.38	0.20	0.53	>0.05
GGT [μkat/L]	0.30	0.22–0.37	0.16	1.31	0.29	0.23–0.38	0.14	1.03	>0.05
CRP [mg/L]	4.50	1.85–5.15	0.02	14.60	5.10	4.40–6.75	2.80	18.60	<0.001
Il-6 [ng/L]	1.50	1.50–2.44	1.50	11.50	1.50	1.50–2.39	1.50	6.24	>0.05
Ca [mmol/L]	2.38	2.32–2.48	2.06	2.65	2.45	2.39–2.53	2.27	2.65	<0.01
Cl [mmol/L]	103	101–106	98	112	104	102–106	100	114	>0.05
K [mmol/L]	4.30	4.02–4.48	3.69	5.05	4.29	4.06–4.45	3.89	5.04	>0.05
Mg [mmol/L]	0.87	0.83–0.91	0.69	1.85	0.83	0.74–0.89	0.41	1.00	<0.01
Na [mmol/L]	136	134–138	132	141	137	136–142	134	143	<0.001
P [mmol/L]	1.03	0.91–1.14	0.72	10.01	1.08	0.94–1.17	0.71	1.36	>0.05

Data are expressed as median (Med) and interquartile range (IQR); Min, minimum; Max, maximum; T-C, total cholesterol; TG, triglycerides; GLU, glucose; UA, uric acid; ALP, alkaline phosphatase; ALT, alanine aminotransferase; AST, aspartate aminotransferase; GGT, gamma-glutamyl transferase; CRP, C-reactive protein; Il-6, interleukin-6; Ca, calcium; Cl, chloride; K, potassium; Mg, magnesium; Na, sodium; P, phosphorus.

**Table 4:** Pre–post intervention changes in terms of LDL/HDL subfractions and CVD risk (*n* = 55).

Parameters	Pre				Post				*p*-Value
	Med	IQR	Min	Max	Med	IQR	Min	Max	
LDL [mmol/L]	3.32	2.70–4.13	1.74	6.31	3.48	2.78–4.04	1.62	7.05	>0.05
VLDL [mmol/L]	0.93	0.78–1.16	0.54	1.66	1.01	0.81–1.18	0.57	9.15	>0.05
IDL-A [mmol/L]	0.75	0.60–0.85	0.26	1.24	0.75	0.52–0.88	0.13	1.24	>0.05
IDL-B [mmol/L]	0.41	0.31–0.52	0.08	0.78	0.47	0.34–0.54	0.13	1.29	>0.05
IDL-C [mmol/L]	0.49	0.39–0.61	0.18	1.03	0.54	0.42–0.70	0.16	1.01	>0.05
LDL-1 [mmol/L]	1.03	0.76–1.18	0.44	1.47	0.98	0.72–1.13	0.21	1.73	>0.05
LDL-2 [mmol/L]	0.28	0.16–0.44	0.05	1.32	0.28	0.16–0.43	0.00	1.22	>0.05
LDL-3 [mmol/L]	0.00	0.00–0.05	0.00	0.39	0.00	0.00–0.08	0.00	0.44	>0.05
LDL3-7 [mmol/L]	0.00	0.00–0.05	0.00	0.47	0.00	0.00–0.08	0.00	0.57	>0.05
Mean LDL size [nm]	27.40	27.20–27.40	26.40	27.60	27.30	27.10–27.40	26.10	27.70	>0.05
HDL [mmol/L]	1.64	1.42–1.85	1.10	2.81	1.72	1.50–1.94	1.11	2.98	>0.05
HDL-1 [mmol/L]	0.13	0.08–0.16	0.03	0.39	0.13	0.10–0.18	0.00	0.41	>0.05
HDL-2 [mmol/L]	0.21	0.16–0.26	0.03	0.47	0.21	0.16–0.26	0.03	0.44	>0.05
HDL-3 [mmol/L]	0.16	0.13–0.23	0.03	0.41	0.18	0.14–0.23	0.03	0.34	>0.05
HDL-4 [mmol/L]	0.21	0.18–0.25	0.05	0.47	0.21	0.16–0.23	0.05	0.39	>0.05
HDL-5 [mmol/L]	0.16	0.13–0.18	0.08	0.28	0.16	0.13–0.18	0.10	0.28	>0.05
HDL-6 [mmol/L]	0.28	0.23–0.31	0.13	0.41	0.31	0.26–0.34	0.13	0.49	<0.05
HDL-7 [mmol/L]	0.10	0.08–0.10	0.05	0.13	0.10	0.08–0.13	0.05	0.16	<0.001
HDL-8 [mmol/L]	0.10	0.08–0.13	0.05	0.16	0.13	0.08–0.16	0.08	0.21	<0.01
HDL-9 [mmol/L]	0.10	0.08–0.13	0.03	0.16	0.13	0.08–0.16	0.03	0.21	<0.01
HDL-10 [mmol/L]	0.16	0.10–0.26	0.03	0.52	0.18	0.13–0.21	0.03	0.65	>0.05
Small [mmol/L]	0.36	0.27–0.51	0.13	0.80	0.41	0.30–0.52	0.13	0.88	>0.05
Intermediate [mmol/L]	0.75	0.65–0.83	0.39	1.29	0.78	0.70–0.85	0.54	1.32	>0.05
Large [mmol/L]	0.49	0.34–0.67	0.10	1.16	0.52	0.44–0.65	0.08	1.09	>0.05
Small/Large	0.77	0.43–1.20	0.22	6.50	0.80	0.50–1.23	0.25	12.39	>0.05
LDL/HDL	2.07	1.61–2.57	0.84	4.51	1.95	1.46–2.62	0.88	4.64	>0.05
TG/HDL	0.66	0.46–0.91	0.28	2.26	0.55	0.41–0.82	0.24	2.12	>0.05
T-C/HDL	2.20	1.85–2.80	0.08	4.90	2.01	1.74–2.89	0.97	4.81	>0.05

Data are expressed as median (Med) and interquartile range (IQR); Min, minimum; Max, maximum; LDL, low-density lipoprotein; VLDL, very low-density lipoprotein; IDL, intermediate-density lipoprotein; HDL, high-density lipoprotein; TG, triglycerides; T-C, total cholesterol.

**Table 5:** Post-intervention changes in biochemical parameters adjusted for total cholesterol.

	TC < 5.0 mmol/L (*n* = 20)					TC > 5.0 mmol/L (*n* = 35)					Inter-Group
	Pre		Post			Pre		Post			
Parameters	Med	IQR	Med	IQR	*p*-Value	Med	IQR	Med	IQR	*p*-Value	*p*-Value
T-C [mmol/L]	4.54	4.210–4.72	4.64	4.12–5.40	>0.05	6.02	5.57–6.67	5.97	5.78–6.66	>0.05	<0.001
TG [mmol/L]	0.79	0.67–1.08	0.82	0.62–1.11	>0.05	1.21	0.89–1.72	1.10	0.89–1.70	>0.05	<0.01
GLU [mmol/L]	5.15	4.85–5.45	5.35	5.15–5.70	>0.05	5.20	4.80–5.58	5.30	5.00–5.68	>0.05	>0.05
UA [μmol/L]	238	206–292	254	201–282	>0.05	261	228–329	258	222–306	>0.05	>0.05
ALP [μkat/L]	1.13	0.93–1.50	1.05	0.88–1.38	>0.05	1.18	1.04–1.56	1.14	0.95–1.40	>0.05	>0.05
ALT [μkat/L]	0.22	0.18–0.35	0.19	0.16–0.35	>0.05	0.28	0.21–0.33	0.25	0.19–0.33	>0.05	>0.05
AST [μkat/L]	0.32	0.28–0.35	0.32	0.27–0.37	>0.05	0.33	0.30–0.40	0.36	0.29–0.38	>0.05	>0.05
GGT [μkat/L]	0.29	0.20–0.36	0.28	0.21–0.41	>0.05	0.31	0.25–0.37	0.30	0.25–0.38	>0.05	>0.05
CRP [mg/L]	4.35	2.20–5.15	4.90	3.95–6.20	>0.05	4.60	1.67–5.15	5.40	4.55–7.28	<0.05	>0.05
Ca [mmol/L]	2.36	2.29–2.41	2.41	2.38–2.46	>0.05	2.39	2.32–2.49	2.51	2.41–2.55	<0.05	<0.01
Cl [mmol/L]	103	102–108	105	103–107	>0.05	103	101–105	103	102–105	>0.05	>0.05
K [mmol/L]	4.22	4.04–4.49	4.29	3.99–4.44	>0.05	4.32	4.01–4.46	4.30	4.08–4.45	>0.05	>0.05
Mg [mmol/L]	0.87	0.84–0.90	0.79	0.72–0.86	<0.05	0.87	0.82–0.93	0.85	0.76–0.91	>0.05	>0.05
Na [mmol/L]	136	136–137	137	136–141	<0.05	136	135–138	137	136–142	>0.05	>0.05
P [mmol/L]	1.05	0.97–1.15	1.09	0.96–1.16	>0.05	1.02	0.90–1.14	1.04	0.94–1.19	>0.05	>0.05

Data are expressed as median (Med) and interquartile range (IQR); T-C, total cholesterol; TG, triglycerides; GLU, glucose; UA, uric acid; ALP, alkaline phosphatase; ALT, alanine aminotransferase; AST, aspartate aminotransferase; GGT, gamma-glutamyl transferase; CRP, C-reactive protein; Ca, calcium; Cl, chloride; K, potassium; Mg, magnesium; Na, sodium; P, phosphorus.

**Table 6:** Post-intervention changes in LDL/HDL subfractions and CVD risk adjusted for total cholesterol.

	TC < 5.0 mmol/L (*n* = 20)					TC > 5.0 mmol/L (*n* = 35)					Inter-Group
	Pre		Post			Pre		Post			
Parameters	Med	IQR	Med	IQR	*p*-Value	Med	IQR	Med	IQR	*p*-Value	*p*-Value
LDL [mmol/L]	2.64	1.97–2.86	2.84	2.06–3.01	>0.05	3.70	3.38–4.55	3.87	3.36–4.47	>0.05	<0.001
VLDL [mmol/L]	0.71	0.65–0.83	0.83	0.71–0.97	>0.05	1.09	0.93–1.24	1.14	0.94–1.29	>0.05	>0.05
IDL-A [mmol/L]	0.62	0.53–0.74	0.63	0.47–0.75	>0.05	0.83	0.70–0.98	0.78	0.62–0.96	>0.05	<0.01
IDL-B [mmol/L]	0.31	0.26–0.40	0.40	0.27–0.47	>0.05	0.47	0.37–0.54	0.52	0.39–0.63	>0.05	<0.01
IDL-C [mmol/L]	0.39	0.32–0.43	0.41	0.39–0.48	>0.05	0.57	0.49–0.67	0.65	0.52–0.77	>0.05	<0.001
LDL-1 [mmol/L]	0.75	0.63–0.99	0.80	0.65–0.94	>0.05	1.11	0.99–1.25	1.03	0.83–1.31	>0.05	<0.01
LDL-2 [mmol/L]	0.16	0.13–0.18	0.18	0.10–0.30	>0.05	0.36	0.22–0.60	0.31	0.21–0.51	>0.05	<0.01
LDL-3 [mmol/L]	0.00	0.00–0.00	0.00	0.00–0.04	>0.05	0.03	0.00–0.11	0.00	0.00–0.09	>0.05	>0.05
LDL3-7 [mmol/L]	0.00	0.00–0.00	0.00	0.00–0.04	>0.05	0.03	0.00–0.11	0.00	0.00–0.09	>0.05	>0.05
LDL size [nm]	27.40	27.40–27.50	27.35	27.20–27.50	>0.05	27.20	27.10–27.40	27.30	26.93–27.40	>0.05	>0.05
HDL [mmol/L]	1.52	1.41–1.70	1.66	1.43–1.90	>0.05	1.69	1.42–1.87	1.75	1.55–1.98	>0.05	<0.05
HDL-1 [mmol/L]	0.13	0.08–0.16	0.13	0.10–0.18	>0.05	0.13	0.08–0.18	0.16	0.10–0.18	>0.05	>0.05
HDL-2 [mmol/L]	0.21	0.17–0.26	0.23	0.21–0.26	>0.05	0.18	0.16–0.25	0.21	0.14–0.28	>0.05	>0.05
HDL-3 [mmol/L]	0.18	0.14–0.23	0.18	0.17–0.21	>0.05	0.16	0.13–0.23	0.16	0.13–0.23	>0.05	>0.05
HDL-4 [mmol/L]	0.21	0.18–0.23	0.21	0.18–0.23	>0.05	0.21	0.16–0.26	0.21	0.16–0.25	>0.05	>0.05
HDL-5 [mmol/L]	0.16	0.14–0.18	0.16	0.16–0.18	>0.05	0.16	0.13–0.18	0.16	0.13–0.18	>0.05	>0.05
HDL-6 [mmol/L]	0.26	0.23–0.31	0.28	0.26–0.31	>0.05	0.28	0.24–0.34	0.31	0.28–0.36	>0.05	>0.05
HDL-7 [mmol/L]	0.08	0.08–0.10	0.10	0.08–0.13	>0.05	0.10	0.08–0.10	0.11	0.10–0.13	<0.05	>0.05
HDL-8 [mmol/L]	0.08	0.08–0.12	0.10	0.08–0.14	>0.05	0.10	0.10–0.13	0.13	0.10–0.18	>0.05	>0.05
HDL-9 [mmol/L]	0.08	0.06–0.10	0.10	0.05–0.14	>0.05	0.10	0.10–0.13	0.13	0.08–0.18	>0.05	>0.05
HDL-10 [mmol/L]	0.10	0.05–0.17	0.14	0.09–0.17	>0.05	0.21	0.16–0.28	0.21	0.16–0.23	>0.05	<0.05
Small [mmol/L]	0.27	0.19–0.35	0.38	0.22–0.44	>0.05	0.47	0.34–0.54	0.44	0.36–0.56	>0.05	<0.05
Intermediate	0.71	0.65–0.82	0.76	0.69–0.80	>0.05	0.78	0.60–0.83	0.80	0.69–0.92	>0.05	>0.05
Large [mmol/L]	0.52	0.41–0.63	0.54	0.48–0.67	>0.05	0.47	0.34–0.69	0.49	0.36–0.64	>0.05	>0.05
Small / Large	0.55	0.31–0.90	0.60	0.39–0.84	>0.05	0.88	0.51–1.51	0.90	0.51–1.37	>0.05	>0.05
LDL/HDL	1.66	1.28–2.05	1.55	1.18–2.01	>0.05	2.24	1.69–2.78	2.21	1.56–2.74	>0.05	<0.01
TG/HDL	0.53	0.36–0.74	0.45	0.38–0.73	>0.05	0.72	0.55–1.14	0.59	0.48–1.02	>0.05	<0.05
T-C/HDL	1.86	1.60–2.21	1.82	1.43–2.08	>0.05	2.60	2.03–3.19	2.40	1.91–3.06	>0.05	<0.01

Data are expressed as median (Med) and interquartile range (IQR); LDL, low-density lipoprotein; VLDL, very low-density lipoprotein; IDL, intermediate-density lipoprotein; HDL, high-density lipoprotein; TG, triglycerides; T-C, total cholesterol.

**Table 7:** Spearman correlation matrix of LDL and HDL subfractions with lipid profile and cardiovascular risk indicators.

Parameters	T-C [mmol/L]	TG [mmol/L]	LDL/HDL	T-C/HDL
VLDL	0.806 ***	0.715 ***	0.479 ***	0.55 ***
IDL-C	0.827 ***	0.548 ***	0.547 ***	0.563 ***
IDL-B	0.528 ***	0.357 ***	0.325 ***	0.415 ***
IDL-A	0.496 ***	0.016	0.389 ***	0.279 **
LDL	0.852 ***	0.563 ***	0.876 ***	0.774 ***
LDL-1	0.655 ***	0.360 ***	0.680 ***	0.563 ***
LDL-2	0.609 ***	0.455 ***	0.462 ***	0.510 ***
LDL3-7	0.413 ***	0.25 **	0.288 **	0.326 ***
HDL	0.154	−0.183	−0.600 ***	−0.606 ***
HDL-1	−0.047	−0.316 ***	−0.368 ***	−0.474 ***
HDL-2	−0.208 *	−0.453 ***	−0.6 ***	−0.678 ***
HDL-3	−0.164	−0.425 ***	−0.591 ***	−0.674 ***
HDL-4	−0.067	−0.390 ***	−0.555 ***	−0.629 ***
HDL-5	−0.208 *	−0.401 ***	−0.656 ***	−0.696 ***
HDL-6	0.147	0.100	−0.198 *	−0.280 **
HDL-7	0.199 *	0.245 **	−0.132	−0.090
HDL-8	0.253 **	0.215 *	−0.181	−0.062
HDL-9	0.320 ***	0.301 **	−0.114	−0.01
HDL-10	0.479 ***	0.315 ***	0.143	0.274 **
Small HDL	0.444 ***	0.323 ***	0.023	0.164
Intermediate HDL	−0.016	−0.229 *	−0.540 ***	−0.608 ***
Large HDL	−0.160	−0.432 ***	−0.558 ***	−0.657 ***

* *p* < 0.05; ** *p* < 0.01; *** *p* < 0.001. VLDL, very low-density lipoprotein; IDL, intermediate-density lipoprotein; LDL, low-density lipoprotein; HDL, high-density lipoprotein; T-C, total cholesterol; TG, triglycerides.

## Data Availability

The data presented in this study are available on request from the corresponding author due to privacy.

## Abbreviations

The following abbreviations are used in this manuscript:

ALP	Alkaline phosphatase
ALT	Alanine transaminase
AST	Aspartate aminotransferase
BMR	Basal metabolic rate
Ca	Calcium
Cl	Chloride
CRP	C-reactive protein
CVD-RF	Cardiovascular disease risk factor
GGT	Gamma-glutamyl transferase
GLU	Glucose
HC	Hip circumference
HDL	High-density lipoprotein
IDL	Intermediate-density lipoprotein
IQR	Interquartile range
K	Potassium
LDL	Low-density lipoprotein
Mg	Magnesium
Na	Sodium
P	Phosphorus
T-C	Total cholesterol
TG	Triglycerides
UA	Uric acid
VLDL	Very low-density lipoprotein
WC	Waist circumference
